# Supplementary material for: Crotamine/siRNA Nanocomplexes for Functional Downregulation of Syndecan-1 in Renal Proximal Tubular Epithelial Cells
Source: Pharmaceutics. 2023 May 23;15(6):1576. doi: 10.3390/pharmaceutics15061576 (PMC10302291; doi:10.3390/pharmaceutics15061576)
Supplement: Supplementary file 1 [file pharmaceutics-15-01576-s001.zip › pharmaceutics-2222276-supplementary.pdf]

Supplementary Data

Materials

Crotamine produced by chemical synthesis was purchased from Smartox Biotechnology (Saint-Egrève, France) to be independent from natural sources and to guarantee standardized compound quality. The synthetic crotamine is characterized as conserving structure and biological activities similar to native peptide [1]. Crotamine was labeled with the Alexa Fluor™ 555 NHS Ester (ThermoFisher Scientific Inc., Waltham, MA, USA), strictly following the instruction of the manufacturer, as described [2]. We used the siRNA-targeting syndecan-1 mRNA GCCGCAATTGTGGCTACTAA, Allstars Negative Control siRNA and fluorescent-labeled scrambled siRNA AF488 (Qiagen, the Netherlands). All other reagents, when not specified in the text, were of analytical grade and were mainly purchased from Sigma-Aldrich Inc. (St. Louis, MO, USA).

Methods

Interaction of crotamine with siRNA in agarose gel evaluated by gel shift assay

The electrophoresis mobility shift assay (EMSA) was used to evaluate the formation of nanocomplexes after the incubation of the chemically synthesized crotamine with siRNA. Samples with crotamine-siRNA molar ratio (nM) of 1:1, 10:1, 50:1, 100:1, and 200:1 were prepared as previously described [3]. The samples were mixed with 6x sample buffer (8 mM Tris-HCl, 0.2 mM EDTA, 50% glycerol, 0.01% (v/w) bromophenol blue), applied on 1% agarose gel in TAE buffer (40 mM Tris, 20 mM acetic acid, 1 mM EDTA) with ethidium bromide, at 65V, for 30 min. After the electrophoresis, the gel was analyzed under ultraviolet light to check the mobility of siRNA molecules stained by the ethidium bromide. The gel was also stained with Coomassie Blue dye (40% methanol, 10% acetic acid, 2.5% (w/v) Coomassie Brilliant Blue R) to visualize crotamine. The

percentage of siRNA complexed with crotamine was calculated by ImageJ software, considering the fluorescence intensity of the gel band correspondent to non-complexed naked siRNA (0% complexed), and the complete absence of siRNA gel band at the expected area of the gel as 100% complexed. The assay was performed two times independently.

Characterization of crotamine-siRNA nanocomplexes by dynamic light scattering (DLS)

The size of crotamine-siRNA nanocomplex was analyzed by DLS using the ZetaSizer ZS ZEN3600 (Malvern Instruments, Malvern, UK), essentially following the manufacture's protocol for this instrument. Measurements were performed in 40 μL cuvettes, at 25°C, for 25 min for each reading. The effect of the ratio variation in the formed nanocomplex was evaluated with a fixed concentration of crotamine and varying the amount of siRNA. Crotamine-siRNA ratios of 10:1, 50:1 and 100:1 (in nM) were tested here. The assay was performed 3 times independently for each sample.

Electron microscopy

The morphology of the prepared crotamine-siRNA was examined by transmission electron microscopy (TEM) with a negative staining with 2% phosphotungstic acid (PTA) [4] using Talos™ F200i S/TEM (ThermoFisher Scientific Inc., Waltham, MA, USA). The assay was performed 3 times independently.

Stability of naked siRNA and crotamine/siRNA nanocomplexes in the presence of serum

The ability of crotamine to protect siRNA from degradation by serum components was analyzed by agarose gel electrophoresis. Crotamine-siRNA nanocomplexes (at a molar ratio of 50:1) were incubated with an equal volume of fetal bovine serum (FBS) (final concentration of 50% v/v) at 37°C for 2 h. After the addition of 0.5% sodium dodecyl sulfate (SDS) to concomitantly inhibit serum RNases and displace siRNA from the complex [5]. Then, the samples were mixed with 6× sample buffer, applied to 1% agarose gel with ethidium bromide and TAE buffer for electrophoresis at 65 V for 30 min. After electrophoresis, the gel was analyzed under ultraviolet light to observe the integrity of siRNA molecules. The assay was performed two times independently.

Downmodulation by crotamine-siRNA of syndecan-1 overexpression in an *in vitro* cytotoxic model

The downregulation of overexpressed syndecan-1 by the nanocomplexes in a cytotoxic model induced by the anthracycline antibiotic doxorubicin hydrochloride (Sigma-Aldrich) were evaluated by using HK-2 cells. About 100,000 cells/well were plated in 6 × 35 mm plates using appropriate DMEM and Ham's F-12 medium, supplemented with 10 ng/mL human recombinant epidermal growth factor (EGF), and were incubated for 24 h, with 5 μM doxorubicin hydrochloride or cell medium as a negative control. The medium was then refreshed and crotamine-siRNA nanocomplexes, at a molar ratio of 100:1, were added to the cells at a concentration of 3 nM for 24 h. The cells were detached and analyzed by qRT-PCR and by flow cytometry (FACS) to determine gene and protein expression, respectively. The assay was performed three times independently.

Quantitative RT-PCR (qRT-PCR)

RNA was isolated from HK-2 cells using FavorPrep Tissue total RNA Mini Kit (Favorgen, Taiwan). 200 ng of RNA from each sample were used to prepare the cDNA using the QuantiTect Reverse Transcription kit (Qiagen), strictly following the manufacturer's instructions. The cDNA of each sample was added to a reaction mix containing 0.2 μM of each primer and 2× Syber Green (Roche Fast Start Universal Sybr Green Master, Sigma-Aldrich, The Netherlands), completing 10 μL. The designed sequences of syndecan-1 primers pair (Sigma-Aldrich) were: forward, 5'-TACTAATTTGCCCCCTGAAG-3' and reverse, 5'-GATATCTTGCAAAGCACCTG-3'. The housekeeping gene (GAPDH) primers (Sigma-Aldrich, The Netherlands) sequences were: forward, 5'-CATCAAGAAGGTGGTGAAGC-3' and reverse, 5'-ACCACCCTGTTGCTGTAG-3'. The ViiA™ 7 system (ThermoFisher), under the amplification conditions as follows: 95°C - 10 min, 40 cycles of 95°C - 15 s, 60°C - 15 s, 72°C 5 s, was used evaluate the gene expression. The analysis was performed using QuantStudio™ 6 Flex and QuantStudio™ 7 (ThermoFisher) software. The cycle threshold (Ct) values of target gene were divided by the Ct values of the housekeeping gene, used as an internal control for each sample to calculate the relative gene expression.

Flow cytometry assay (FACS)

To analyze syndecan-1 surface expression, properdin binding, and activated C3 deposition, cells were incubated with non-enzymatic cell dissociation solution 1× (C5789 - Sigma®), 600 μL/well, at 37ºC until cells were detached. Then, the cells were transferred to 4.5 mL tubes containing 2 mL cell medium, and after spinning down by centrifugation at 200 ×g, for 5 min at 20ºC, the cells were washed twice with 2 mL medium and incubated with 3 μg/mL properdin (Human Factor P, Millipore), for 30 min, at 37ºC.

After incubation, cells were washed twice with 2 mL medium and incubated with 5% normal human serum as a source of complement from Sigma-Aldrich Inc. (St. Louis, MO, USA) for 45 min at 37 ºC. The cells were then washed twice with 2 mL of ice-cold FACS buffer (1% bovine serum albumin in PBS) and were incubated with rabbit anti-human-properdin [6] (dil. 1:400), or with mouse anti-human-activated C3 recognizing C3b, iC3b, and C3c fragments (Clone bH6, HM2168S, Hycult Biotech, Uden, The Netherlands) (dil. 1:50), or with FITC® mouse anti-human syndecan-1 (which is CD138; Bio-Rad/ AbD Serotec, CA, USA) (dil. 1:100), for 30 min, on ice, in FACS buffer. After, cells were washed twice with 2 mL of ice-cold FACS buffer and incubated for 30 min on ice in FACS buffer with secondary antibodies: goat-anti-rabbit IgG-FITC (Southern Biotech, Birmingham, AL, USA) (dil. 1:100) for properdin staining and goat-a-mouse IgG-FITC (Southern Biotech, Birmingham, AL, USA) (dil. 1:100) for activated C3 and syndecan-1 staining. Thereafter, the cells were washed twice with 2 mL of ice-cold FACS buffer, resuspended in 100 µL of FACS buffer, stained with propidium iodide (PI) to exclude non-viable cells, and kept on ice for FACS analysis in a NovoCyte Quanteon Flow Cytometer™ (Agilent Technologies, Inc, CA, USA). Results are from three independent experiments.

Cryosections

After animal euthanasia, mice tissues were collected, snap frozen in liquid nitrogen, and stored at -80 °C for subsequent histological analysis. Frozen sections (5 - 7 µm thick) were prepared from these mice tissues using a cryostat Microm HM 550 (Thermo Scientific Inc.). The sections were fixed in acetone for 10 min, followed by rehydration in PBS. The sections were then stained with DAPI, before the analysis by confocal microscopy.

Confocal microscopy

Data acquisition was performed in Leica TCS SP8 confocal microscope (Leica Microsystems, Wetzlar, Germany), and Zeiss LSM 780 (Carl-Zeiss AG, Oberkochen, Germany). For HK-2 cells, the parameters used were λ_{Ex} 405 nm/λ_{Em} 420 - 470 nm for DAPI and Hoechst fluorescence, λ_{Ex} 475 - 495 nm/λ_{Em} at 520 - 560 nm for AF488-siRNA, λ_{Ex} 545 nm/λ_{Em} at 590 - 620 nm for AF555-crotamine and λ_{Ex} 650 nm/λ_{Em} at 688 nm for AF647-syndecan-1 and LysoTracker Deep Red, using 63× oil objective. Colocalization was analyzed using the JacoP plugin for ImageJ software based on Mander's coefficient [7]. Venn diagram values represent percentage positive pixels for (overlapping) stainings and were calculated from three distinct images for each experiment.

For kidney sections the parameters used were λ_{Ex} 405 nm/λ_{Em} 420 - 470 nm for DAPI and λ_{Ex} 475 - 495 nm/λ_{Em} at 520 - 560 nm for AF488-siRNA using 100× oil objective.

Results

Incubation of HK-2 cells with doxorubicin for 24 h induced injury in these cells leading to overexpression of syndecan-1, and after cell medium was refreshed, the complexes formed by crotamine and siRNA targeting syndecan-1 [3 nM] at a molar ratio of 100:1 were added and incubated for 24 h. Analysis by real-time PCR of these cells incubated with doxorubicin allowed observing an increased (225%) levels of syndecan-1 mRNA compared to control untreated cells ($p < 0.05$). In addition, the treatment with crotamine/siRNA complexes after the removal of the doxorubicin decreased the syndecan-1 mRNA levels to levels similar to that observed in the control untreated cells, which may indicate a successful suppression of syndecan-1 upregulation or a modulation of syndecan-1 mRNA expression maintaining its physiological levels. Treatment of these

cells with crotamine/siRNA complex, without previous nephrotoxic induction, decreased mRNA levels about 42% compared with control untreated cells ($p < 0.01$; Supplemental Figure S1A).

Flow cytometry analysis confirmed that incubation of cells with doxorubicin increased syndecan-1 protein expression, properdin binding and C3 activation expression by 120% ($p < 0.01$), 118% (non-significant, NS) and 144% (NS), respectively. Cells treated with crotamine/siRNA complexes after the removal of doxorubicin showed a reduction of syndecan-1 expression, properdin binding and C3 activation by 89% ($p < 0.0001$), 52% ($p < 0.001$) and 88% (NS), respectively, while cells treated with crotamine/siRNA complex with no previous nephrotoxic induction showed a lower reduction in expression of syndecan-1, properdin binding and C3 activation by 65% ($p < 0.0001$), 43% ($p < 0.001$) and 56% ($p < 0.05$), respectively, compared to control untreated cells (Supplemental Figure S1B–D).

Supplemental Figure S1

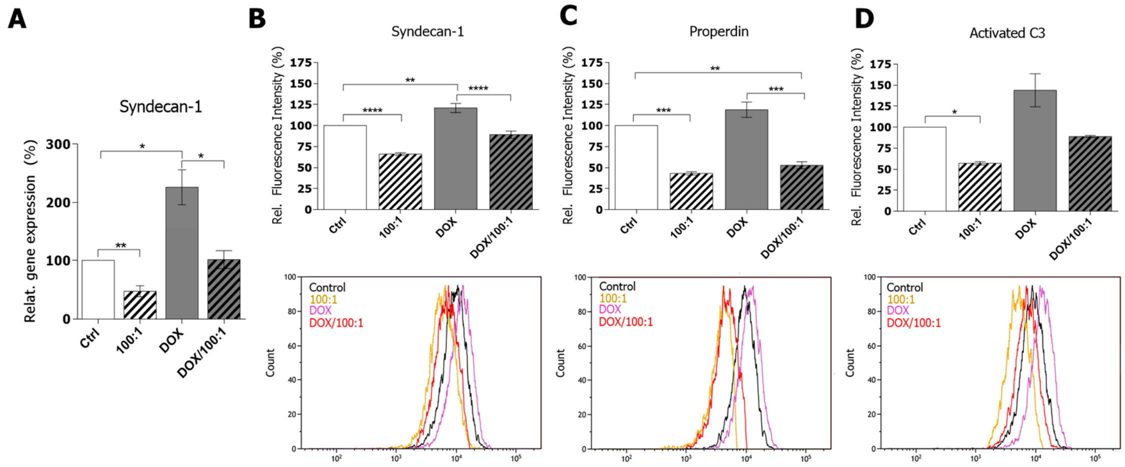

Supplemental Figure S1. Down-modulation of syndecan-1 expression by crotamine-siRNA nanocomplex, properdin binding and C3 complement activation in doxorubicin cytotoxic model in PTEC *in vitro*. qPCR quantification of syndecan-1 gene expression normalized against GAPDH (**A**), flow cytometry analysis of syndecan-1 protein expression and representative flow cytometry experiment below (**B**), flow cytometry analysis of syndecan-1 dependent properdin binding with representative flow cytometry experiment below (**C**), flow cytometry analysis of properdin-mediated activated C3 complement factor and representative flow cytometry experiment below (**D**). Data represented as % relative gene expression for qPCR and % relative fluorescence intensity for flow cytometry (data normalized for the untreated control at 100%) and analyzed by ANOVA and post-hoc Bonferroni test (N = 3, *$p < 0.05$, ** $p < 0.01$, *** $p < 0.001$, ****$p < 0.0001$).

References

1. de Carvalho Porta L, Fadel V, D'Arc Campeiro J, Oliveira EB, Godinho RO, Hayashi MAF. Biophysical and pharmacological characterization of a full-length synthetic analog of the antitumor polypeptide crotamine. *J Mol Med (Berl)* 2020; 98: 1561–1571
2. Hayashi MAF, Oliveira EB, Kerkis I, Karpel RL. Crotamine: a novel cell-penetrating polypeptide nanocarrier with potential anti-cancer and biotechnological applications. *Methods Mol Biol* 2012; 906: 337–352

3. Hayashi MAF, Campeiro JD, Porta LC et al. Crotamine Cell-Penetrating Nanocarriers: Cancer-Targeting and Potential Biotechnological and/or Medical Applications. *Methods Mol Biol* **2020**; *2118*: 61-89

4. Barreto-Vieira DF, Barth OM. Negative and Positive Staining in Transmission Electron Microscopy for Virus Diagnosis. *In*: Shah MM, ed. Microbiology in Agriculture and Human Health. *InTech*, 2015

5. Chen P-C, Hayashi MAF, Oliveira EB, Karpel RL. DNA-interactive properties of crotamine, a cell-penetrating polypeptide and a potential drug carrier. *PLoS One* **2012**; *7*: e48913

6. Gaarkeuken H, Siezenga MA, Zuidwijk K et al. Complement activation by tubular cells is mediated by properdin binding. *Am J Physiol Renal Physiol* 2008; 295: F1397-1403

7. Bolte S, Cordelières FP. A guided tour into subcellular colocalization analysis in light microscopy. *J Microsc* 2006; 224: 213–232
